# Identification of Five Key Genes Involved in Intrinsic Apoptotic Pathway From Yellow Catfish *Pelteobagrus fulvidraco* and Their Transcriptional Responses to High Fat Diet (HFD)

**DOI:** 10.3389/fphys.2019.00921

**Published:** 2019-08-02

**Authors:** Dan-Dan Li, Shi-Cheng Ling, Kun Wu, Zhi Luo

**Affiliations:** ^1^Key Laboratory of Freshwater Animal Breeding, Ministry of Agriculture, Fishery College, Huazhong Agricultural University, Wuhan, China; ^2^Laboratory for Marine Fisheries Science and Food Production Processes, Qingdao National Laboratory for Marine Science and Technology, Qingdao, China

**Keywords:** apoptosis, intrinsic mitochondrial pathway, gene characterization, *Pelteobagrus fulvidraco*, high fat diet

## Abstract

The hypothesis of the present study is that apoptosis through an intrinsic mitochondrial pathway may mediate high fat diet (HFD)-induced changes in the metabolism of *Pelteobagrus fulvidraco*. To this end, we cloned the full-length cDNA sequences of Cycs, Apaf1, Casp9, Casp3a, and Casp3b involved in the mitochondria apoptotic pathway, and explored their mRNA tissue expressions and transcriptional responses to HFD. All of these members shared similar domains to their orthologous vertebrate genes. They were constitutively expressed in all analyzed tissues but varied from tissue to tissue. Compared to the control, HFD up-regulated the mRNA expression of partial genes among these five key genes (Cycs, Apaf1, Casp9, Casp3a, and Casp3b) in mesenteric fat, intestine, ovary and the kidney, indicating the induction of apoptosis in these tissues; in contrast, HFD down-regulated mRNA levels of partial genes among the five key genes (Cycs, Apaf1, Casp9, Casp3a, and Casp3b) in the heart, spleen and gill tissues, indicating the inhibition of apoptosis in these tissues. The present study will facilitate further exploration into the functions of these genes at the molecular level and disclose the critical involvement of these genes against nutrient changes, indicating that processes of apoptosis in various tissues may differentially be modified by HFD.

## Introduction

Apoptosis is a highly regulated and conserved form of programmed cell death for multicellular organisms, which is trigged by a variety of physiological or pathological stimuli (Mu et al., 2010). In mammals, two main pathways triggering apoptosis, the extrinsic/death receptor pathway and the intrinsic/mitochondrial pathway, have been described, respectively (Marsden and Strasser, 2003). For the intrinsic pathway, upon induction of apoptosis, pro-apoptotic proteins can trigger mitochondrial outer membrane permeabilization and release of cytochrome c (Cycs) into the cytosol (Liu et al., 1996). Cytochrome c then binds to apoptotic protease-activating factor-1 (Apaf1) forming the Apaf1/cytochrome c complex (Jiang and Wang, 2000). The complex facilitates the activation of caspase 3 (Casp3) by caspase 9 (Casp9), finally leading to apoptotic cell death (Wang, 2001; Li et al., 2003).

Cycs, Apaf1, Casp9, and Casp3 are the key genes involved in the mitochondrial apoptotic pathway, and their identification was the first important step in characterizing the role and mechanism of apoptosis. During the last few years, the structure, regulation and the function of Cycs, Apaf1, Casp9, and Casp3 in apoptosis had been extensively investigated in mammals (Zou et al., 1997; Reis et al., 2007b; Kumaresan et al., 2016). However, information about their molecular characterization and tissue expression profiles is very limited in fish. At present, Casp9 has been characterized in sea bass (*Dicentrarchus labrax*) (Reis et al., 2007a), common carp (*Cyprinus carpio*) (Gao et al., 2013), yellow croaker (*Pseudosciaena crocea*) (Mu et al., 2010), and striped murrel (*Channa striatus*) (Kumaresan et al., 2016). Casp3 has been investigated in sea bass (Reis et al., 2007b), zebrafish (*Danio rerio*) (Yabu et al., 2001), Atlantic salmon (*Salmo salar*) (Takle et al., 2006), yellow croaker (Li et al., 2011) and striped murrel (Kumaresan et al., 2016), but two Casp3 subtypes (Casp3a and Casp3b) were characterized only in Atlantic salmon (Takle et al., 2006) and medaka *Oryzias latipes* (Naruse et al., 2000). To the best of our knowledge, the sequence information of Cycs and Apaf1 genes still remains unknown in fish. In addition, limited studies explore their constitutive tissue expression profiles in fish, such as Casp9 in sea bass (Reis et al., 2007a) and large yellow croaker (Mu et al., 2010), Casp3 in large yellow croaker (Li et al., 2011).

Apoptotic occurrence can be influenced by dietary composition (Tie et al., 2019; Ye et al., 2019; Yin et al., 2019). Dietary fat plays an important role in providing energy, essential fatty acids (EFAs) and fat-soluble vitamins (Luo et al., 2005), especially for fish, as they have limited abilities in utilizing carbohydrates as an energy source. However, high dietary fat levels is harmful for fish growth and health (Luo et al., 2005). Using mammals and their cell lines, several studies report that HFDs influenced the process of apoptosis, associated with the disruption of mitochondrial function (Moraes et al., 2009). However, in fish, studies involved in the effect of HFD on expression of apoptosis related genes are very scarce.

Yellow catfish *Pelteobagrus fulvidraco*, a freshwater omnivorous teleost, was widely cultured in several Asian countries due to its good filet quality and high market value. However, under intensive aquaculture, dietary fat levels often amount to 10.0%, which inevitably leads to excess fat deposition in the livers and visceral tissues of yellow catfish. Excessive fat deposition will reduce growth performance and a healthy status for this fish species. Since apoptosis is a highly regulated form of programmed cell death and related to a healthy status for organisms, the present working hypothesis is that HFD can influence mRNA expression of genes involved in the mitochondrial apoptotic pathway. To this end, in this study, the full-length cDNA sequences of Cycs, Apaf1, Casp9, Casp3a and Casp3b were cloned and characterized, and their tissue-specific expressions were explored from yellow catfish *P. fulvidraco*, a widely distributed freshwater omnivorous teleost in several Asian countries. The transcriptional responses of Cycs, Apaf1, Casp9, Casp3a, and Casp3b genes to HFD were then investigated in various tissues of *P. fulvidraco*.

## Materials and Methods

Two experiments were conducted. Experiment 1 was conducted to clone the full-length cDNA sequences of Cycs, Apaf1, Casp9, Casp3a and Casp3b, and to investigate their mRNA tissue expression profiles. Experiment 2 evaluated the changes of mRNA levels of Cycs, Apaf1, Casp9, Casp3a, and Casp3b of various tissues in yellow catfish to HFD. We ensured that the experiments performed on animals followed the ethical guidelines of Huazhong Agricultural University and confirm that all experimental protocols were approved by Huazhong Agriculture University.

### Experiment 1: Cloning of Cycs, Apaf1, Casp9, Casp3a, and Casp3b Genes and Exploring Their mRNA Tissue Expression

Yellow catfish *P. fulvidraco* (initial body weight: 16.5 ± 3.4 g, mean ± SEM) were obtained from a local commercial farm, Wuhan, China, and stocked in indoor fiberglass tanks. The culture protocols were similar to those described in our recent publication (Wei et al., 2017). Briefly, yellow catfish (initial body weight: 16.5 ± 3.4 g, mean ± SEM) were maintained in indoor cylindrical fiberglass tanks (300 L water volume) for a 2 week acclimation. All fish were fed a commercial pellet diet with a fat level of 9.7%, twice a day. They were provided with continuous aeration to maintain the dissolved oxygen level near saturation. At the end of the 2 week acclimation, fish were fasted for 24 h and then euthanized with MS-222 (100 mg/L). Liver, muscle, spleen, intestine, gill, mesenteric fat, heart, kidney, and ovary tissues were quickly collected and frozen in liquid nitrogen and stored at −80°C for RNA isolation. RNA isolation, synthesis of cDNAs and cloning of Cycs, Apaf1, Casp9, Casp3a, Casp3b genes were based on the protocols described in our studies (Wei et al., 2017). The quality of total RNA was checked by agarose gel electrophoresis. The concentration and the ratio of OD_260_/OD_280_ of total RNA were determined using a Nanodrop ND-2000 spectrophotometer (Thermo Fisher Scientific, United States) for the determination of OD_260_, OD_280_ and OD_230_ (OD_260_/OD_280_ > 1.8, OD_260_/OD_230_ > 1.5). Degenerate primers (Supplementary Table 1), designed based on the most conserved regions of these fish Cycs, Apaf1, Casp9, Casp3a, and Casp3b sequences available in the GenBank and Ensembl database, were used to amplify partial cDNA fragments. The 3′ and 5′ end sequences were obtained through nested 3′ and 5′ RACE PCR performed with a SMART RACE cDNA Amplification Kit (Clontech, United States) based on the manufacturer’s manual.

The edition of assembled full-length sequences, sequence alignments and percentage of amino acid conservation were similar to those described in our recent publication (Wei et al., 2017). Domains were analyzed by the SMART program^1^ and an online CDD tool at NCBI^2^. The phylogenetic trees were generated through a neighbor-joining (NJ) method with MEGA 5.0 (Tamura et al., 2011) based on the JTT +G model (Jones et al., 1992), and the best-fit model of sequence evolution was obtained by ML model selection. Bootstrap sampling was reiterated 1000 times.

### Experiment 2: Transcriptional Responses of Cycs, Apaf1, Casp9, Casp3a, and Casp3b of Various Tissues of Yellow Catfish Fed HFDs

Two experimental diets were formulated with dietary fish oil/soy-oil (1:1, w/w) supplemented at fat levels of 6% (control fat) and 10% (HFD). Final fat levels were determined to be 11.34 and 15.41% for the control and HFD, respectively. When beginning the feeding experiment, 30 uniform-sized fish (mean initial weight: 3.79 ± 0.16 g) were randomly stocked in each fiberglass tank. Two diet (control and HFD) was assigned to six tanks in a completely randomized design, three replicates for each diet. The fish were fed to apparent satiation twice daily at two equal meals (9:00 and 16:00 h) during the week. The experiment continued for 8 weeks.

At the termination of the feeding study, all fish were fasted for 24 h. They were then anesthetized with tricaine methane sulfonate (MS-222 at 100 mg/L). Three fish per aquarium were collected randomly, and mesenteric fat, intestine, ovary, kidney, heart, spleen and gill tissues were isolated and quickly frozen in liquid nitrogen, and reserved at −80°C for the subsequent analysis for quantitative PCR.

### qPCR Determination

The mRNA levels were assayed by the real-time qPCR method described in our recent publication (Wei et al., 2017). The primer sequences of each gene used in this analysis are given in Supplementary Table 2. The qPCR program included 1 min at 95°C and 40 cycles at 95°C for 5 s, 60°C for 10 s, and 72°C for 30 s. All reactions were performed in duplicate, and each reaction mixture was checked to ensure that it contained a single product of the correct size by agarose gel electrophoresis. As housekeeping gene sequences were not available in *P. fulvidraco*, a set of eight housekeeping genes (18S rRNA, RPL7, β-actin, HPRT, TUBA, B2M, TBP, GAPDH, ELFA, and UBCE) were selected from the literature (Zhao et al., 2011) in order to test their transcription stability. For each control gene, we determined the pairwise variation with all other control genes as the standard deviation of the logarithmically transformed expression ratios and defined the internal control gene stability by measuring the M value as the average pairwise variation of a particular gene with all the other control genes. Genes with the lowest M values have the most stable expression. The relative expression of genes was calculated using the 2^–ΔΔCt^ method (Livak and Schmittgen, 2001) normalizing to the geometric mean of the best combination of two genes as suggested by geNorm (Vandesompele et al., 2002). Prior to the analysis, experiments were performed to check the stability of housekeeping genes, from which GAPDH and HPRT, β-actin and UBCE showed the most stable level of expression in tissues distribution analysis experiment and HFD experiment, respectively, across the experimental conditions.

### Statistical Analysis

Results are presented as mean ± SEM. Prior to statistical analysis, all data were tested for normality of distribution using the Kolmogorov–Smirnov test. The results of the homogeneity of variances among the different tissues were then determined by one-way analysis of variance (ANOVA) and Tukey’s multiple range test. Differences between the control and HFD group were analyzed by Student’s *t*-test for independent samples. The analysis was carried out using the SPSS 20.0 for Windows (SPSS, Michigan Avenue, Chicago, IL, United States), and the minimum significant level was set at 0.05.

## Results

### cDNA Cloning and Sequence Analyses of Five Genes

Using RT-PCR and 3′- and 5′-RACE PCR, we successfully cloned the full-length cDNA sequences of Cycs, Apaf1, Casp9, Casp3a, and Casp3b. The validated cDNA sequences of *P. fulvidraco* cytochrome c (Pf-Cycs, GenBank Accession No. KY053836), *P. fulvidraco* Apaf1 (Pf-Apaf1, GenBank Accession No. KY053839), *P. fulvidraco* caspase 9 (Pf-Casp3b, GenBank Accession No. KY053837), *P. fulvidraco* caspase 3a (Pf-Casp3a, GenBank Accession No. KY072821), *P. fulvidraco* caspase 3b (Pf-Casp3b, GenBank Accession No. KY072822) were 754, 5028, 1544, 1831, and 1225 bp in length, encoding the peptides of 104, 1259, 442, 284, and 285 amino acid residues, respectively (Table 1).

**TABLE 1:** The sequence information of five apoptosis related genes from *P. fulvidraco.*

**Genes**	**Accession No.**	**5′ UTR (bp)**	**ORF (bp)**	**3′ UTR (bp)**	**Full length (bp)**	**Protein (aa)**
Cycs	KY053836	81	315	358	754	104
Apaf1	KY053839	127	3780	1121	5028	1259
Casp9	KY053837	33	1329	182	1544	442
Casp3a	KY072821	219	855	757	1831	284
Casp3b	KY072822	86	858	281	1225	285

The pair-wise amino acid sequence comparison of five genes between different species are shown in Table 2. The Cycs in *P. fulvidraco* shared high identity (83.7–99%) with the corresponding Cycs orthologous genes from other fish species, amphibians and mammals. The amino acid sequences of *P. fulvidraco* Cycs, Apaf1, caspase 9, caspase 3a, and caspase 3b were similar to those from other fish and mammals, exhibiting 51.2–88.3, 38.1–83.3, 57.2–85.5, and 55.1–77.9% amino acid sequence identities, respectively.

**TABLE 2:** Amino acid sequence identity of five apoptosis related genes between *P. fulvidraco* and other species (%).

**Genes**	***Ictalurus punctatus***	***Danio rerio***	***Xenopus tropicalis***	***Mus musculus***	***Homo sapiens***
Cycs	99	99	87.5	90.4	83.7
Apaf1	88.3	76.5	51.2	56	56.5
Casp9	83.3	72.4	38.1	48.5	48.4
Casp3a	85.5	74.5	57.2	58.5	57.7
Casp3b	77.9	65.8	55.1	59.4	56.9

*Accession numbers as follows (the order is *Ictalurus punctatus*, *Danio rerio*, *Xenopus tropicalis*, *Mus musculus*, and *Homo sapiens*): Cycs (XP_017338026.1, NM_001002068.1, ENSXETP00000049267, JF919281.1, NM_018947.5); Apaf1 (XM_017485104, NM_131608.1, XM_012959843.2, NM_001042558.1, ENSP00000448165); Casp9 (XM_017487601, NM_001007404.2, NM_001123463, XM_017319928.1, ENSP00000330237); Casp3a (XP_017317002, NM_131877.3, NM_001127428.1, NM_001284409.1, XM_011532301.1); Casp3b (NM_001201081, XM_005173075.4, NM_001127428.1, NM_001284409.1, XM_011532301.1).*

**FIGURE 1:**
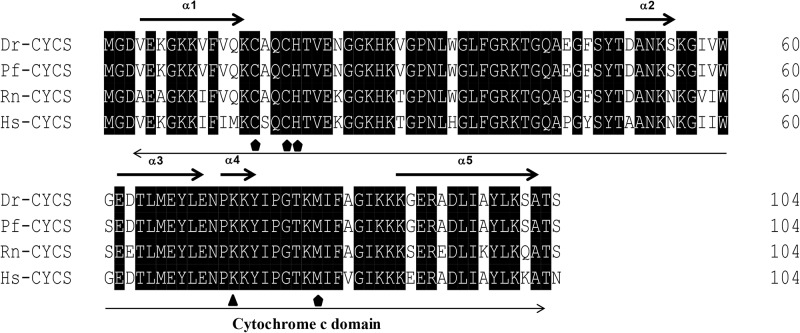
Multiple amino acid sequence alignments of Cycs genes of *Pelteobagrus fulvidraco* and other species. Accession numbers are ENSRNOP00000041521, NM_018947.5, NM_001002068.1, KY053836 for *Rattus norvegicus* (Rn), *Homo sapiens* (Hs), *Danio rerio* (Dr), and *Pelteobagrus fulvidraco* (Pf), respectively. Arrows above and below the sequences represent α-helices and the Cytochrome c domain, respectively. Residues below symbols (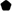) were identified as a specific site, including the interactive sites with heme and iron ion. Residues below symbol (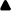) means the modified sites of trimethyllysine.

**FIGURE 2:**
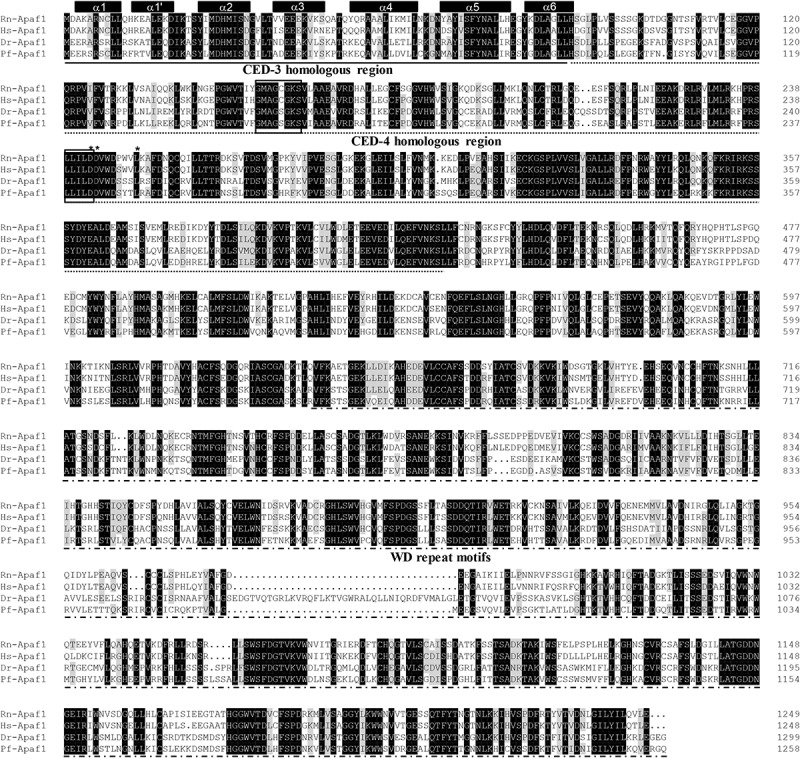
Multiple amino acid sequence alignment of Apaf1 genes from *P. fulvidraco* and other species. Accession numbers were ENSRNOP00000041521, ENSP00000448165, NM_131608.1, KY053839 for *Rattus norvegicus* (Rn), *Homo sapiens* (Hs), *Danio rerio* (Dr), and *Pelteobagrus fulvidraco* (Pf), respectively. The CED-3 and CED-4 homologous region and WD repeat motifs indicate in a continuous and discontinuous line. Apaf1 CARD (caspase recruitment domain) contains seven short α-helices which are represented by cylinders on top of the sequence alignment. The Walker’s A- and B-box consensus sequences for nucleotide binding sites at amino acid residues 153–160 and 238–242 are boxed. Asterisks denote the amino acid residues that are known to be important for CED-4 function. Deep and light shadows represent homology of 100 and 75%, respectively.

**FIGURE 3:**
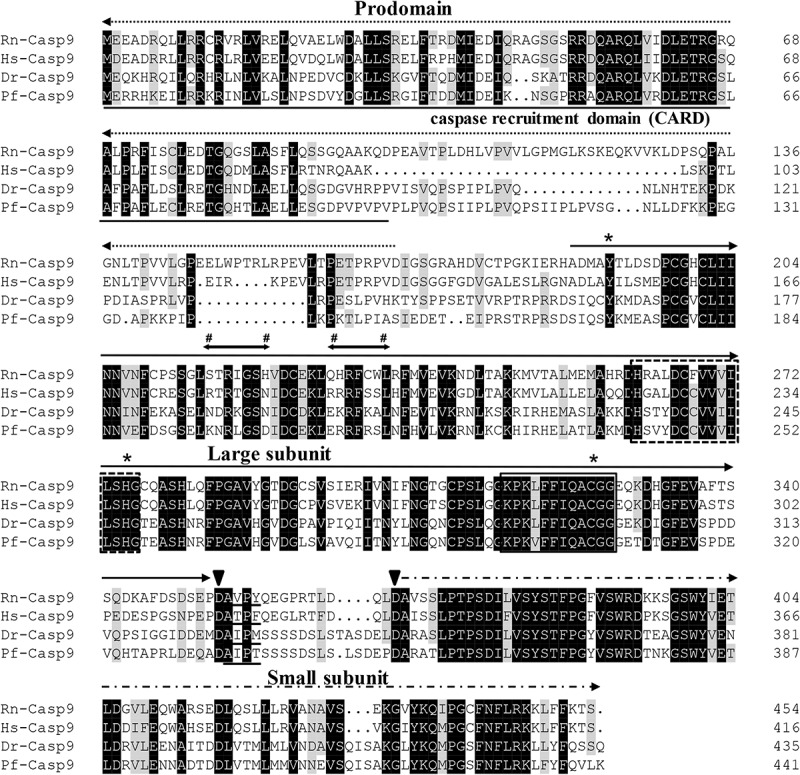
Multiple amino acid sequence alignments of Casp9 genes from *P. fulvidraco* and other species. Accession numbers as follows [the order is *Rattus norvegicus* (Rn), *Homo sapiens* (Hs), *Danio rerio* (Dr), and *Pelteobagrus fulvidraco* (Pf)]: Casp9 (ENSRNOP00000017972, ENSP00000330237, NM_001007404.2, KY053837). The putative cleavage site at aspartic acid residues (Asp333 and Asp351), which separates the large subunit (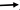) from the small subunit (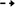) are indicated by arrowheads. The prodomain is indicated by a dashed line with an arrowhead (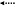). The caspase family histidine (residues 242–256) and cysteine active sites (residues 296–307) are boxed in a discontinuous or continuous line, respectively. The first 100 amino acids (caspase recruitment domain, underlined) are a putative N-terminal CARD which are presumably required to bind to Apaf1. Residues below symbols (#) indicate the consensus and putative Akt phosphorylation motifs. The A-X-P-X motifs are underlined. The conserved ‘Tyr,’ ‘His,’ and ‘Cys’ residues are indicated with an asterisk (^*^). The prodomain regions comprise conserved ‘Arg’ and ‘Leu’ residues. Deep and light shadows represent homology of 100 and 75%, respectively.

**FIGURE 4:**
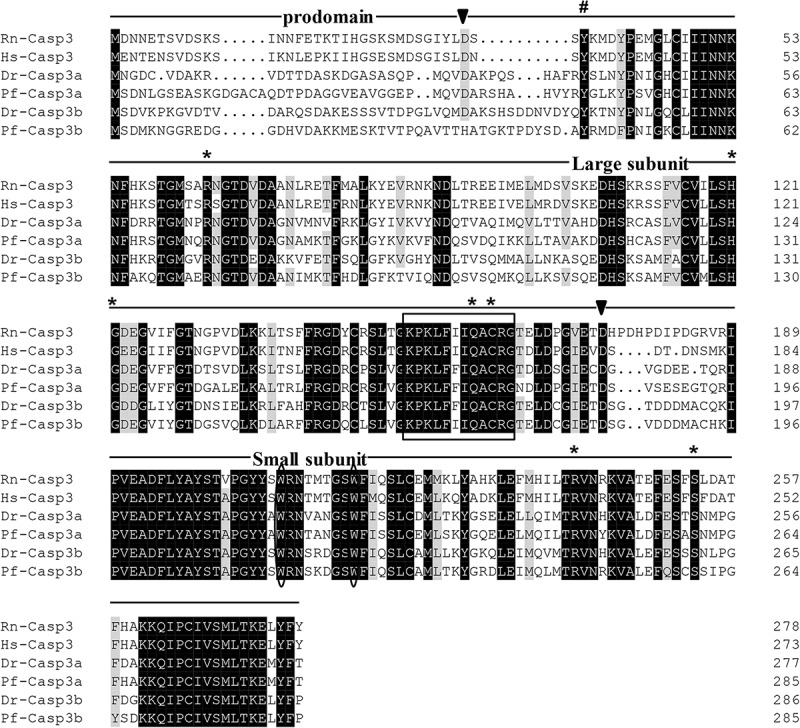
Multiple amino acid sequence alignments of Casp3 genes from *P. fulvidraco* and other species. Deduced amino acid sequences were obtained from [*Rattus norvegicus* (Rn), *Homo sapiens* (Hs), *Danio rerio* (Dr), and *Pelteobagrus fulvidraco* (Pf)] and Accession numbers are shown in order as follows: ENSRNOP00000014096, XM_011532301.1, NM_131877.3, KY072821, XM_005173075.4, KY072822. The putative cleavage sites at aspartic acid residues, which seperates caspase3 into prodomain, large subunit and small subunit, are indicated by arrowheads. The cysteine active sites are boxed. The two tryptophan residues that undergo rearrangement when Casp3 is activated are presented with an oval frame. The conserved tyrosine residues below symbols (#) is suggested to play a critical role in downstream apoptosis activity. The Several residues known to be critical in the caspase3 catalytic and binding pocket are indicated by an asterisk.

The protein sequences of *P. fulvidraco* Cycs possessed highly conserved Cycs domain, including several interactive sites with heme and iron ion and one modified site of trimethyllysine, composing of five α-helices (α1–α5) (Figure 1). The *P. fulvidraco* Apaf1 embraced all the characteristic features of Apaf1, including the CED-3 and CED-4 homologous regions, the seven short α-helices identified in Apaf1 CARD, the Walker’s A- and B-box consensus sequences for nucleotide binding sites, and the WD repeat motifs are indicated (Figure 2). The predicted amino sequence of Casp9 contained a similar architecture than mammals, including a prodomain, a large subunit, a small subunit, the putative cleavage sites, a caspase recruitment domain (CARD), consensus and putative Akt phosphorylation motifs, A-X-P-X motifs, caspase family histidine and cysteine active sites along with the conserved active residues ‘His’ and ‘Cys.’ Furthermore, the conserved ‘Arg’ and ‘Leu’ residues in prodomain and the conserved ‘Tyr’ were also found in *P. fulvidraco* (Figure 3). Casp3 (Casp3a and 3b) contained a similar structure with mammals, including a prodomain, a large subunit, a small subunit, the putative cleavage sites, and cysteine active sites. Several residues known to be critical in the Casp3 catalytic and binding pocket were found in *P. fulvidraco*. The two tryptophan residues and tyrosine residues were also found in *P. fulvidraco* and other vertebrates (Figure 4). In Casp9, two putative cleavage sites at aspartic acid residues separates the large subunit and small subunit, while the large subunit is contiguous with the small subunit in Casp3.

### Phylogenetic Analysis

**FIGURE 5:**
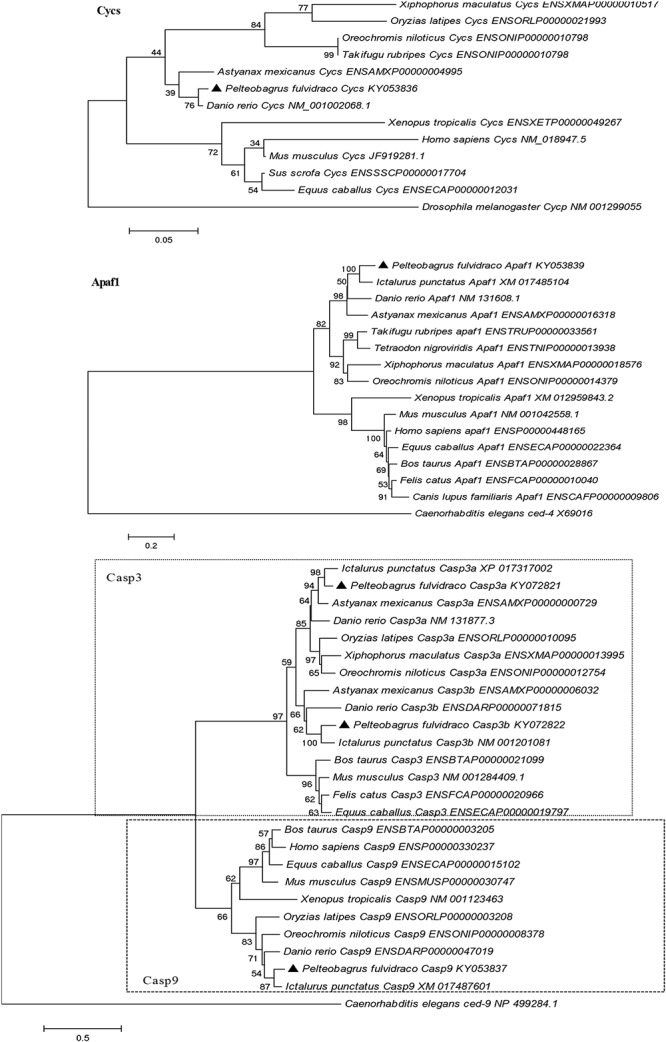
Phylogenetic tree based on the protein sequences of Cycs, Apaf1, Casp3a, Casp3b, and Casp9 from *P. fulvidraco* and other vertebrate species using the neighbor-joining (NJ) method in MEGA 5.0 (Tamura et al., 2011) based on the JTT+G model (Jones et al., 1992). Branch support values represent a percentage of 1000 bootstrap replicates.

A phylogenetic analysis based on the amino acid sequences of Cycs, Apaf1, Casp9, Casp3a, and Casp3b from *P. fulvidraco* and other vertebrate species are shown in Figure 5. According to the phylogenetic analysis, all teleost cytochrome c formed an independent cluster, while amphibian and mammalian cytochrome c formed another cluster. *P. fulvidraco* Cycs were grouped with *Astyanax mexicanus* and *Danio rerio*, the same members of Ostariophysi. They then formed a clade with the species of Acanthomorphata (*Takifugu rubripes*, *Oryzias latipes*, *Xiphophorus maculatus*, *Oreochromis niloticus*). A similar topology was also observed in phylogenetic trees of Apaf-1. The phylogenetic analysis showed that all Casp3 and Casp9 genes clustered with the corresponding sequences of vertebrates. Casp3 clades together constituted the caspase 3 subfamily, which is clearly separated from Casp9 clade. All teleost Casp3a and Casp3b formed a cluster, whereas mammalian Casp3 formed another clade. Moreover, on the basis of the tree topologies, there appeared to be a fish-specific gene duplication which may result in the generation of an additional Casp3 member in *P. fulvidraco* and other fishes (Casp3a and Casp3b).

### mRNA Expression Patterns of Cycs, Apaf1, Casp9, Casp3a, and Casp3b Genes Among Nine Tissues

**FIGURE 6:**
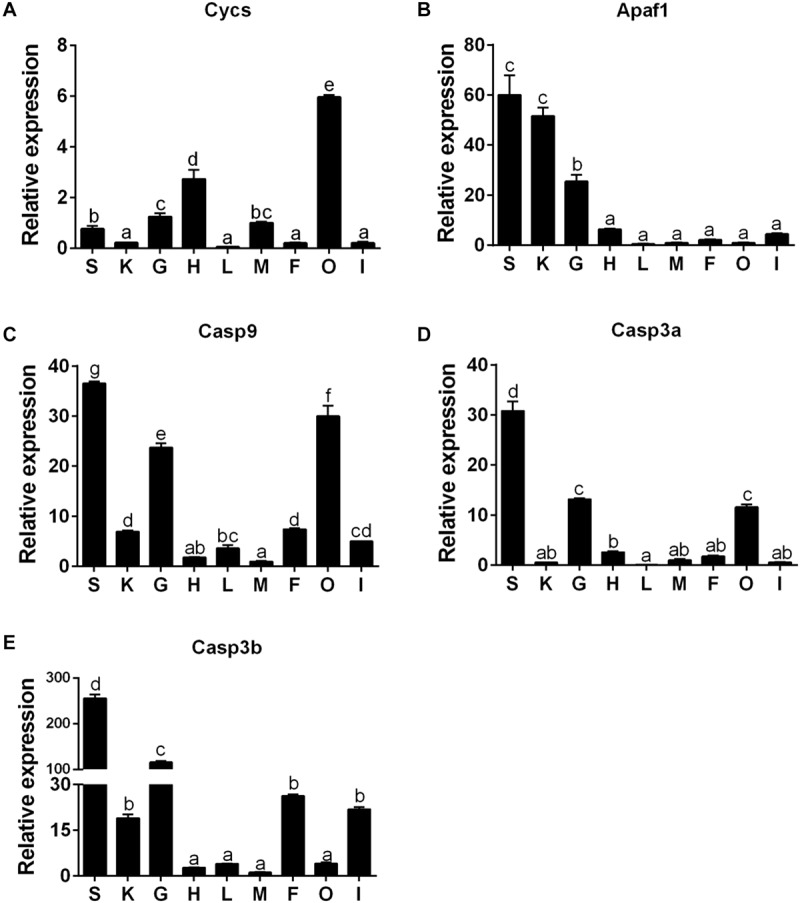
The tissue-specific expression of five apoptosis related genes across spleen (S), kidney (K), gill (G), heart (H), liver (L), muscle (M), mesenteric fat (F), ovary (O), and intestine (I) of *P. fulvidraco* via quantitative PCR (Q-PCR) (**A:** Cycs; **B:** Apaf1; **C:** Casp9; **D:** Casp3a; **E:** Casp3b). Data (mean ± SEM, *n* = 6) were expressed relative to expression of housekeeping gene (GAPDH and HPRT). Expression of genes in muscle was regarded as the relative expression 1. Bars with different letters indicate significant differences among nine tissues (*P* < 0.05).

The Cycs mRNA was the highest in ovary tissues, followed by the heart, gill, muscle, and spleen tissues, but lowest in the kidney, liver, mesenteric fat, and intestine tissues (Figure 6A). The mRNA of Apaf1 was predominantly expressed in spleen and kidney tissues, followed by gill tissues, and lowest in heart, intestine, mesenteric fat, muscle, liver, and ovary tissues (Figure 6B). The mRNA of Casp9 was highest in spleen tissues, followed by ovary, gill, mesenteric fat, kidney, intestine, liver tissues, and the lowest in the heart and muscle tissues (Figure 6C). The Casp3a mRNA expression was predominant in spleen tissues, followed by gill, ovary and heart tissues, and the lowest in mesenteric fat, muscle, kidney, intestine, and the liver tissues (Figure 6D). For the Casp3b mRNA levels, the values were highest in the spleen tissues, followed by gill, mesenteric fat, intestine and the kidney tissues, and showed no significant differences in other tissues (Figure 6E).

### Transcriptional Responses of Cycs, Apaf1, Casp9, Casp3a, and Casp3b of Various Tissues in Yellow Catfish Fed HFDs

**FIGURE 7:**
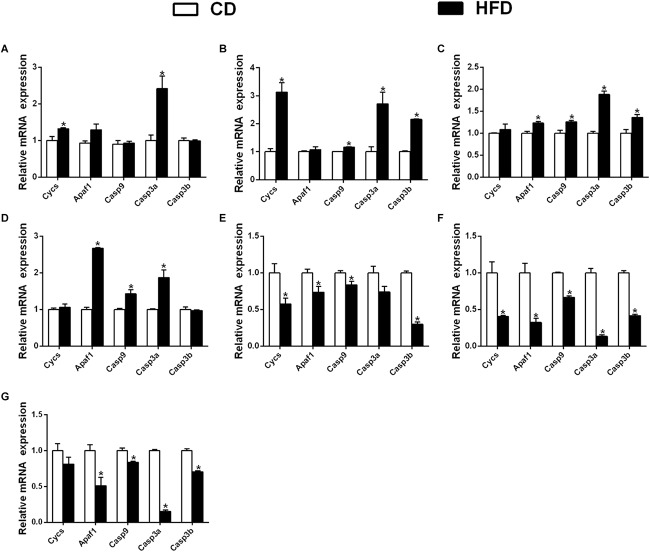
Effect of an HFD on mRNA expression of gene i mitochondria-caspase pathway in *P. fulvidraco* across different tissues (**A:** mesenteric fat; **B:** intestine; **C:** ovary; **D:** kidney; **E:** heart; **F:** spleen; **G:** gill). ^a,b,c^Values are mean ± SEM (*n* = three replicate tanks, and three fish were sampled from each tank); Data are expressed relative to the expression of the housekeeping genes (β-actin and UBCE). Asterisk indicates significant differences between the control and HFD (*t*-test, *p* < 0.05). CD, control diet; HFD, high fat diet.

In the mesenteric fat tissues, compared to the control, a HFD up-regulated the mRNA expression of Cycs and Casp3a and had no significant effect on the expression of Apaf1, Casp9, and Casp3b (Figure 7A); in the intestine tissues, a HFD upregulated the expressions of Cycs, Casp9, Casp3a and Casp3b, but Apaf1 expression showed no significant change (Figure 7B). In ovary tissues, a HFD upregulated the mRNA expression of Apaf1, Casp9, Casp3a and Casp3b significantly, and the expression of Cycs remained relatively constant (Figure 7C). In kidney tissues, a HFD significantly upregulated the mRNA expression of Apaf1, Casp9 and Casp3a, but did not significantly influence the expression Cycs and Casp3b (Figure 7D).

In heart tissues, compared with the control, a HFD down-regulated the mRNA expression of Cycs, Apaf1, Casp9 and Casp3b, but did not significantly influence the expression of Casp3a (Figure 7E); in spleen tissues, the expression of Cycs, Apaf1, Casp9, Casp3a, and Casp3b were down-regulated in a HFD (Figure 7F). In gill tissues, a HFD significantly down regulated the mRNA expressions of Apaf1, Casp9, Casp3a and Casp3b, but did not significantly influence Cycs mRNA expression (Figure 7G).

## Discussion

In the present study, we successfully cloned the full-length cDNA sequences of Cycs, Apaf1, Casp9, Casp3a, and Casp3b from *P. fulvidraco*. To the best of our knowledge, this is the first report involving cDNA sequence information and mRNA tissue expression profiles of Cycs and Apaf1, and their transcription responses of the five genes to a HFD in fish.

Our study indicated that the putative protein sequences of *P. fulvidraco* Cycs and Apaf1 embraced all the characteristic features of their corresponding parts in mammals, indicating that they might have a similar function to those in mammals. The predicted amino acid sequence of caspase 9 contained a putative CARD motif, followed by the large (p20) and small (p10) subunit, in agreement with other studies (Gao et al., 2013; Kumaresan et al., 2016). Furthermore, the conserved ‘Arg’ and ‘Leu’ residues in prodomain and the conserved ‘Tyr,’ as reported by Kumaresan et al. (2016), were also found in *P. fulvidraco*. The characteristic Casp9 pentapeptide active-site QACGG is conserved in other fish (Gao et al., 2013) and also located in the large subunit (Reis et al., 2007a). In the present study, the deduced Casp3 (Casp3a and 3b) in yellow catfish is highly homologous with the Casp3 of other species, including a putative prodomain followed by a large and a small subunit (Yabu et al., 2001; Reis et al., 2007b; Li et al., 2011). The Casp3 sequence retains the motifs that are functionally important, such as the pentapeptide active-site motif (QACRG) and the putative cleavage sites at the aspartic acids, in agreement with those in large yellow croaker and sea bass (Reis et al., 2007b; Li et al., 2011). In addition, the present study also cloned two Casp3 isoforms in yellow catfish, in agreement with reports in medaka *Oryzias latipes* (Naruse et al., 2000) and Atlantic salmon (Takle et al., 2006). However, in other fish species, the Casp3 gene exists as a single copy gene (Reis et al., 2007b; Li et al., 2011).

The successful cloning of the five key genes enabled us to study their constitutive and diet-induced tissue expression profiles. However, to the best of our knowledge, this is the first report involved in mRNA constitutive tissue expression profiles of Cycs, Apaf1 and Casp3b in fish, which limited our comparison with other fish. The present study showed that mRNAs of the five genes were constitutively expressed in all tested tissues, indicating that they participated in many physiological functions in these tissues. However, their mRNA expression varied with the tissues, indicating their tissue-specific roles. On the other hand, we found that the Cycs mRNA was the highest in ovary tissues, followed by heart, gill, muscle and spleen tissues, but lowest in kidney, liver, mesenteric fat, and intestine tissues. In shrimp, Hu and Yao (2016) reported the ubiquitous expression of Cycs in most examined tissues of shrimp *Litopenaeus vannamei*, but predominantly in muscle tissues, followed by intestine, gill, and heart tissues. The present study indicated that the mRNA of Apaf1 was predominantly expressed in spleen and kidney tissues, followed by gill, and lowest in heart, intestine, mesenteric fat, muscle, liver, and ovary tissues. In mammals, Walke and Morgan (2000) reported that Apaf1 transcript was detected in many tissues, and the highest level of expression was seen in spleen and lung tissues. We also found that the mRNA of Casp9 was highest in spleen tissues, followed by ovary, gill, mesenteric fat, kidney, intestine, liver, and the lowest in heart and muscle tissues, in partial agreement with several other studies. For example, Mu et al. (2010) pointed out that large yellow croaker Casp9 was constitutively expressed in all tissues examined with heart tissues containing the highest levels, and intestine and muscle tissues containing the lowest levels. In sea bass, Reis et al. (2007a) observed the highest expression levels of Casp9 in heart and liver tissues, and low expression in spleen and kidney tissues. Kumaresan et al. (2016) reported that Casp9 was highly expressed in trunk kidney tissues followed by head kidney tissues of *C. striatus*. The present study found that two Casp3 subtypes, were differentially expressed in various tissues. For example, the Casp3a mRNA expression was predominant in spleen tissues, followed by gill, ovary and heart tissues, the lowest in mesenteric fat, muscle, kidney, intestine, and liver tissues; for the Casp3b mRNA levels, the values were highest in spleen tissues, followed by gill, mesenteric fat, intestine and kidney tissues, and showed no significant differences in other tissues. The differences in expression patterns among yellow catfish caspases suggested that each caspase might differ functionally, as suggested by Takle et al. (2006). In large yellow croaker, Li et al. (2011) reported that Casp3 was constitutively expressed in various tissues, with higher levels in blood, heart, kidney, spleen, intestine and gill tissues, but lower levels in liver and muscle tissues. In *C. striatus*, the maximum expression of Casp3 is observed in spleen tissues (Kumaresan et al., 2016). In contrast, in sea bass, Casp3 mRNA was expressed in all tissues examined, albeit at low levels (Reis et al., 2007b).

In fish, the dynamic mRNA expression of apoptosis-related genes has been used as a marker at an earlier point in the apoptotic cascade (Gao et al., 2013), and their increased mRNA expression has been described in cells under apoptosis (Chiang et al., 2001; Yakovlev et al., 2001). The present study indicated that effects of a HFD on the mRNA expression of genes in intrinsic mitochondria pathways were tissue dependent. Generally speaking, two trends were observed: in the mesenteric fat, intestine, ovary and kidney tissues, a HFD induced mRNA levels of several genes among these five key genes; however, in the heart, spleen and gill tissues, a HFD down-regulated mRNA expression of partial genes among these five key genes. At present, we do not know the exact reason, since this is the first study exploring the changes of their mRNA expression of genes involved in the mitochondrial apoptotic cascade of various tissues in fish fed an HFD. Apoptosis is one of major types of cell death, and previous studies demonstrate that diets could influence the expression of apoptotic genes. For example, Camargo et al. (2017) showed that long-term consumption of low-fat, high-complex carbohydrate diets (LFHCC) and LFHCC n-3 diets increased the expression of Casp3 in the adipose tissue. Olivo and Hilakivi-Clarke (2005) showed that an HFD inhibited apoptosis in rat mammary glands. An HFD induced apoptosis in skeletal muscle (Sishi et al., 2011) and liver tissues (Wang et al., 2008). Moraes et al. (2009) observed that an HFD induced the hypothalamic expression of Apaf1 and Casp9. In livers of rats fed HFD compared to those in the control, there was higher levels of cleaved Casp3 (Wang et al., 2008). Espe et al. (2015) pointed out that enhanced activation of caspase 3 was associated with the oxidative stress from Atlantic salmon. Li et al. (2017) found that Zn increased hepatic transcriptional levels of cytochrome C, caspase 3a and caspase 3b in yellow catfish, which indicated that Zn induced mitochondrial-mediated apoptosis. Yin et al. (2019) found that the expression of caspase 9 significantly increased with an increasing oxidative degree of fish oil in largemouth bass. Thus, the reduced apoptotic mRNA levels in the HFD group in heart, spleen, and gill tissues will inhibit apoptosis via mitochondrial apoptotic pathways, which may result in the impairment of the capacity for regeneration and repair of these tissues and organs, as suggested by Ye et al. (2019). Increased mRNA levels of these apoptotic genes in mesenteric fat, intestine, ovary, and kidney tissues indicate that an HFD activates the mitochondrial apoptotic pathway and induces apoptosis in these organs, as suggested by Li et al. (2017). Therefore, our result indicates that an HFD differentially influences mRNA expression of the genes of various tissues in intrinsic mitochondrial pathways, indicating that different mechanisms for regulating intrinsic apoptotic pathways existed in various tissues. Thus, our data highlights the complexity and inconsistency of the HFD-induced apoptotic effects in different tissues. Furthermore, we speculate that the different mRNA expressions of those apoptosis related genes might be attributable to the HFD-induced changes of fat metabolism in various tissues of *P. fulvidraco*.

## Conclusion

In summary, we characterized the full-length cDNA sequences of five genes involved in the intrinsic mitochondrial apoptotic pathway (Cycs, Apaf1, Casp9, Casp3a, and Casp3b) and explored their tissue expression profiles in *P. fulvidraco*, which will facilitate further exploration into their functions at the molecular level. HFD-induced expression studies disclose the critical involvement of these genes against nutrient changes, indicating that the processes of apoptosis in various tissues may be modified by an HFD.

## Data Availability

All datasets generated for this study are included in the manuscript and/or the Supplementary Files.

## Ethics Statement

We assured that the experiments performed on animals followed the ethical guidelines of Huazhong Agricultural University and confirmed that all experimental protocols were approved by Huazhong Agriculture University.

## Author Contributions

ZL and D-DL designed the experiment. D-DL conducted the experiment and sample analysis, with the help of S-CL and KW. D-DL and ZL analyzed the data. D-DL drafted the manuscript. ZL revised the manuscript. All the authors approved the manuscript.

## Conflict of Interest Statement

The authors declare that the research was conducted in the absence of any commercial or financial relationships that could be construed as a potential conflict of interest.

## Footnotes

1 http://www.smart.embl-heidelberg.de/

2 http://www.ncbi.nlm.nih.gov/Structure/cdd/wrpsb.cgi

## Abbreviations

Apaf1: apoptotic peptidase activating factor-1
Casp3a: caspase 3a
Casp3b: caspase 3b
Casp9: caspase 9
Cycs: cytochrome c
ELFA: translation elongation factor
GAPDH: glyceraldehyde-3-phosphate dehydrogenase
HPRT: hypoxanthine-guanine phosphoribosyltransferase
RPL7: ribosomal protein L7
SEM: standard error of mean
TBP: TATA-box-binding protein
TUBA: tubulin alpha chain
UBCE: ubiquitin-conjugating enzyme
