# Supplementary material for: Identification of Five Key Genes Involved in Intrinsic Apoptotic Pathway From Yellow Catfish Pelteobagrus fulvidraco and Their Transcriptional Responses to High Fat Diet (HFD)
Source: Front Physiol. 2019 Aug 2;10:921. doi: 10.3389/fphys.2019.00921 (PMC6687843; doi:10.3389/fphys.2019.00921)
Supplement: Supplementary file 1 [file Table_1.DOC]

**Supplementary Table 1.** Nucleotide sequences of the primers used for the cDNA cloning from *P. fulvidraco*.

| Primers | Sequence (5'-3') |
| --- | --- |
| **Primers for partial fragment** | |
| Cycs-F | GTCCAGAAGTGCGCMCAGTG |
| Cycs-R | GATGCCAACAAGAGCAAAGG |
| Apaf1-F | AAAGAATGYAAAGGCTCTCC |
| Apaf1-R | GGAGGATAYATCAAGTGGTGG |
| Casp9-F | GACTGCTGTGTGGTBRTCAT |
| Casp9-R | TGTAYAAACAGATGCCTGG |
| Casp3a-F | ATCRTCATCAACAACAAGAACTT |
| Casp3a-R | GGCTCHTGGTTCATBCAGTC |
| Casp3b-F | TCATHATCAACAACAAGAAYTT |
| Casp3b-R | CGDGTCAACCRCAAGGTGGC |
| **Primers for 3'-RACE PCR** | |
| 3'GS-Cycs-O | GACGGMGTGAACTGGGGSCGSATC |
| 3'GS-Cycs-I | TTCCGTAGGGTYGGAGGTAAVAGG |
| 3'GS-Apaf1-O | CAGCAGATYGGAGAYGARCTGGA |
| 3'GS-Apaf1-I | CAGACGGTYCACCCHCACGGYTT |
| 3'GS-Casp9-O | TTTAAYGACMWGATCGATGGHTTAG |
| 3'GS-Casp9-I | TAAGAAGGGGTCAACTAKACMGGTAG |
| 3'GS-Casp3a-O | AGCTGTSTGACCCCARCCAYAA |
| 3'GS-Casp3a-I | TCGCGKTGGTGAGMCGGGGTCAA |
| 3'GS-Casp3b-O | CTACTGCTCCAGGTTATTATGCTT |
| 3'GS-Casp3b-I | GCTGACTAAGTATGGCCGTGAT |
| 3'RACE Outer | GCTCATTTWCMAGGCGCTGACTCA |
| 3'RACE Inner | GGGTMGGAGGAACGASAGACTAGGT |
| **Primers for 5'-RACE PCR** | |
| 5'GS-Cycs-O | GCCAGTCTTACGTCCAAACAGA |
| 5'GS-Cycs-I | TGTGCTTGCCACCGTTCT |
| 5'GS-Apaf1-O | CGATACGGTCGCTGATTACA |
| 5'GS-Apaf1-I | GACCCCACAGAACAGACAGAAC |
| 5'GS-Casp9-O | TGCCCATTGAGGTAGTTAGTGAT |
| 5'GS-Casp9-I | AAGCGATTATGACTTGCCTCA |
| 5'GS-Casp3a-O | CAGTGGCTGTGGTCATCCTT |
| 5'GS-Casp3a-I | AAAAGTCTTCATGGCGTTTCC |
| 5'GS-Casp3a-O | GGTCCTCTTGGGATACTGATTTTA |
| 5'GS-Casp3a-I | TTTGCAGCATCAACATCAGTTC |
| 5'RACE Outer | CTAATACGACTCACTATAGGGC |

**Notes:** K-G/T; M-A/C; R-A/G; S-G/T; Y-C/T; B-G/T/C; D-G/A/T; H-A/T/C; V-G/A/C; N-A/T/G/C.

**Supplementary Table 2.** Primers used for real-time quantitative PCR analysis

| **Genes** | **Forward primers**  **(5'-3')** | **Reverse primers**  **(5'-3')** | **Size** | **Accession**  **no.** |
| --- | --- | --- | --- | --- |
| β-actin | GGACTCTGGTGATGGTGTGA | CTGTAGCCTCTCTCGGTCAG | 138 | EU161066 |
| RPL7 | GGCAAATGTACAGGAGCGAG | GCCTTGTTGAGCTTGACGAA | 199 | KP938522 |
| HPRT | ATGCTTCTGACCTGGAACGT | TTGCGGTTCAGTGCTTTGAT | 181 | KP938523 |
| TUBA | TCAAAGCTGGAGTTCTCGGT | AATGGCCTCGTTATCCACCA | 135 | KP938526 |
| B2M | GCTGATCTGCCATGTGAGTG | TGTCTGACACTGCAGCTGTA | 186 | KP938520 |
| UBCE | TCAAGAAGAGCCAGTGGAGG | TAGGGGTAGTCGATGGGGAA | 150 | KP938524 |
| TBP | AGCAAAGAGTGAGGAGCAGT | ACTGCTGATGGGTGAGAACA | 170 | KP938525 |
| GAPDH | TTTCAGCGAGAGAGACCCAG | ATGACTCTCTTGGCACCTCC | 132 | KP938521 |
| 18S rRNA | AGCTCGTAGTTGGATCTCGG | CGGGTATTCAGGCGAGTTTG | 196 | KP938527 |
| ELFA | GTCTGGAGATGCTGCCATTG | AGCCTTCTTCTCAACGCTCT | 151 | KU886307 |
| Cycs | CTTTTCCTACAC  GGATGCCA | TCGCCCTTCTTC  TTGATGC | 135 | KY053836 |
| Apaf1 | ACCGCCAAATA  GCAACCTG | CTGCTCCTCGTG  CTCAACAT | 101 | KY053839 |
| Casp9 | CACAGCACCAA  GGCTAGATGA | TCCTGGAAATGTT  GAGTAGGACA | 145 | KY053837 |
| Casp3a | CTCATTTGTTTG  CGTGTTGC | CACTGGGATTTTC  ATCGAATTATCT | 223 | KY072821 |
| Casp3b | CTTCATCATTCA  GGCTTGTCG | GCATAATAACCTGGAGCAGTAGAG | 141 | KY072822 |

**Abbreviations:** Apaf1, apoptotic peptidase activating factor 1; Casp3a, caspase3a; Casp3b, caspase3b; Casp9, caspase9; Cycs, cytochrome c, somatic; ELFA, translation elongation factor. GAPDH, Glyceraldehyde-3-phosphate dehydrogenase; HPRT, hypoxanthine-guanine phosphoribosyltransferase; RPL7, ribosomal protein L7; TBP, TATA-box-binding protein; TUBA, tubulin alpha chain; UBCE, ubiquitin-conjugating enzyme. The primers of Cycs, Apaf1, Casp9, Casp3a and Casp3b genes were highlighted by boldface.
